# Validating the Effectiveness of the Patient-Centered Cancer Care Framework by Assessing the Impact of Work System Factors on Patient-Centered Care and Quality of Care: Interview Study With Newly Diagnosed Cancer Patients

**DOI:** 10.2196/53053

**Published:** 2024-04-24

**Authors:** Safa Elkefi, Onur Asan

**Affiliations:** 1 School of Nursing Columbia University New York, NY United States; 2 School of Systems and Enterprises Stevens Institute of Technology Hoboken, NJ United States

**Keywords:** cancer, communication, trust, satisfaction, technology, workload, work system factors

## Abstract

**Background:**

Patients with cancer who have recently been diagnosed have distinct requirements compared to cancer survivors. It is crucial to take into account their unique needs to ensure that they make informed decisions and are receptive to the care provided.

**Objective:**

This study suggested a framework titled Effectiveness of Patient-Centered Cancer Care that considers the needs of newly diagnosed patients with cancer and related work system factors. This study investigated how work system factors influence the perceptions of patient-centered care, quality of care, and associated outcomes among newly diagnosed patients with cancer. Patient-centered care is defined in terms of workload and communication considerations, whereas the quality of care is assessed through indicators such as trust in physicians, satisfaction with care, and perceptions of technology.

**Methods:**

This study used qualitative data collected through interviews with newly diagnosed patients with cancer (N=20) right after their first visits with their physicians. Thematic analysis was conducted to validate the 5 hypotheses of the framework, mapping the interactions among quality of care, patient-centered care, and work system factors.

**Results:**

We found that workload and patient-centered communication impact the quality of care and that the work system elements impact the patient-centeredness (workload and communication) and the quality of care (trust in physicians, satisfaction with care, and perception of technology use).

**Conclusions:**

Qualitatively validating the proposed Effectiveness of Patient-Centered Cancer Care framework, this study demonstrated its efficacy in elucidating the interplay of various factors. The framework holds promise for informing interventions geared toward enhancing patients’ experiences during their initial visits after diagnosis. There is a pressing need for heightened attention to the organizational design, patient processes, and collaborative efforts among diverse stakeholders and providers to optimize the overall patient experience.

## Introduction

### Background

Improving the quality of care (QOC), coordination, and quality of life are essential goals of chronic care [1]. Patient-centered care (PCC) is one of the approaches used to assure the primacy of the individual’s health and life goals in their care management [1]. In recent years, the concept of having the person be the driving force in their health care decisions has evolved and gained momentum, and it is now largely considered the gold standard for health care worldwide [1,2].

The initial physician visits after a cancer diagnosis are a critical period in which patients face a range of challenges that can significantly disrupt their lives. Symptoms of the disease and the overwhelming decisions related to treatment can pose a threat to their physical, cognitive, and emotional well-being [3]. Patients often struggle to comprehend medical information and express frustration with prolonged waiting periods for prognoses and follow-ups [3]. This can lead to psychosocial concerns, including high levels of distress, emotional strain, uncertainty surrounding mortality, and disruptions to social life [3]. The cognitive and emotional burdens can be overwhelming, potentially leading to nonadherence to treatment plans [4].

PCC approaches are considered crucial for the delivery of high-quality care to patients. However, there is considerable ambiguity concerning the exact meaning of the term and the optimal method for measuring the process and outcomes of PCC [5]. Despite the concept’s popularity in the past 30 years, there has been a slight argument of perspective in the literature about the definition of PCC [5]. It has been an evolving concept, originally presented by Balint [6], who described patient-centered medicine as understanding the patient as a unique human being, whereas for Levenstein et al [7], it is an approach in which the “physician enters the patient’s world to see the illness through his eyes.” In 1998, Delbanco et al [8] developed a self-described utopian vision for a patient-centered health care system called People Power. The relationship is supported by “computer-based guidance and communication systems.” Don Berwick, a former administrator for the Centers for Medicare and Medicaid Services, has popularized the slogan Delbanco and his group adopted, “Nothing about me without me,” acknowledging that PCC is not always evidence based. In his 2009 *Health Affairs* article, he emphasized that PCC relates to one’s set of decisions and choices of circumstances and relationships in health care.

This concept has received increased attention since the 2001 Institute of Medicine report, “Crossing the Quality Chasm” [9], where health care quality and system-of-care improvement efforts were linked to the 6 core values: safe, effective, efficient, patient centered, timely, and equitable. Since then, myriad clinical, policy, and research initiatives have been launched to promote the study, advancement, and implementation of PCC. Research later presented 8 primary dimensions of the PCC model (respect of values, physical comfort, coordination and integration of care, information and education, access to care, involvement of family and friends, and transition and continuity) [10]. In 2015, the World Health Organization released its framework on “people-centered health services” [11], emphasizing a focus on a system that adopts individuals’, careers’, families’, and communities’ perspectives into a trusted health care system.

PCC frameworks have proved to change the behavior of patients with cancer as they successfully engage the patient by incorporating his biopsychosocial support system into care delivery and ensuring sustainable development [12]. Involving patients with cancer meaningfully in the processes and responding to their emotions as part of PCC adoption have been linked to better health outcomes, more trust, and better engagement of the patient in their care [13]. Thus, to evaluate the effectiveness of PCC initiatives, the cognitive perception of patients with cancer needs to be studied in relation to their behavior within the care settings (eg, trust, satisfaction, anxiety, and engagement). On the other hand, achieving high-quality care is a complex pursuit in any setting, especially for cancer care. Improving the patient journey requires an integrated system of care and productive interactions among many system levels. By understanding the work system components, the design and integration of tasks, technology, and clinical processes can be reviewed to better support the needs of individuals while optimizing system performance. A supportive work environment and a highly engaged workforce correlate with improved quality of PCC and hospital performance [14]. Case managers, navigators, quality officers, and administrators may track patient outcomes at the population level. A study conducted in 2017 on postdiagnosis treatment communication with patients with cancer highlighted the importance of coordination among specialists, primary care, and other people involved in the care processes with patients to deliver necessary care as problems in coordination can lead to fragmentation in health outcomes and processes. However, existing initiatives and care-planning processes face barriers to adoption and implementation. To sum up, tools and initiatives designed to improve health care delivery through PCC need to be inspired by systems engineering principles as recommended by the Institute of Medicine and the National Academy of Engineering to identify, develop, and sustain best practices informed by the needs of survivors, caregivers, clinicians, organizations, and communities [13].

Due to the complex nature of the health care system, it remains hard to provide patients with care that meets their expectations without accounting for the work system in which they are receiving the care services [15]. However, to our knowledge, no framework focuses on PCC from a systems perspective. Human factors engineering interventions need to take into account issues across the whole system (system approach) with macro-ergonomic considerations, including organizational factors, to be more likely to significantly impact QOC. The Systems Engineering Initiative for Patient Safety (SEIPS) model of work system and patient safety, for example, emphasizes the principle of “balance” and focuses on system interactions that need to be considered to make significant progress in health care quality, linking the work system factors to health outcomes [16]. In addition, although many studies have focused on the workload of physicians and staff, no study has focused on the workload of patients with cancer. In this qualitative study, we explored the impact of work system factors on newly diagnosed patients with cancer’s perceptions of PCC and QOC and the impact of PCC on the QOC outcomes among newly diagnosed patients with cancer following a suggested conceptual framework.

### Theoretical Background

#### Overview

The framework built was inspired by different human factors models such as social cognitive theory [17], which conceptualizes the behavior of a person as a result of mental, personal, and social and environmental factors, therefore we considered behavior as a sum of a patient’s perceptional cognitive input (patient-centeredness perception) and the response. Our patient-centered effectiveness components were inspired by the patient-centered communication in cancer care that defines communication through 6 functions [18] and the technology acceptance model. The technology acceptance model links technology perception to the attitude of the user toward the perceived usefulness and ease of use and the external variables [19,20]. The last model that inspired our framework is the SEIPS [21]. From a sociotechnical perspective, patients’ experience, especially with chronic diseases, is a function of many coordination challenges [22]. Therefore, we need to go beyond the typical focus on a patient’s single health care encounter and understand a patient’s journey from a broader perspective through their interactions with other stakeholders in a system where not only patients and physicians are actors but the work system and the tools used are also important impactors of the perceptions and decision-making processes. Thus, we look at the systems’ factors impacting patients’ perception of patient-centeredness.

**Figure 1:**
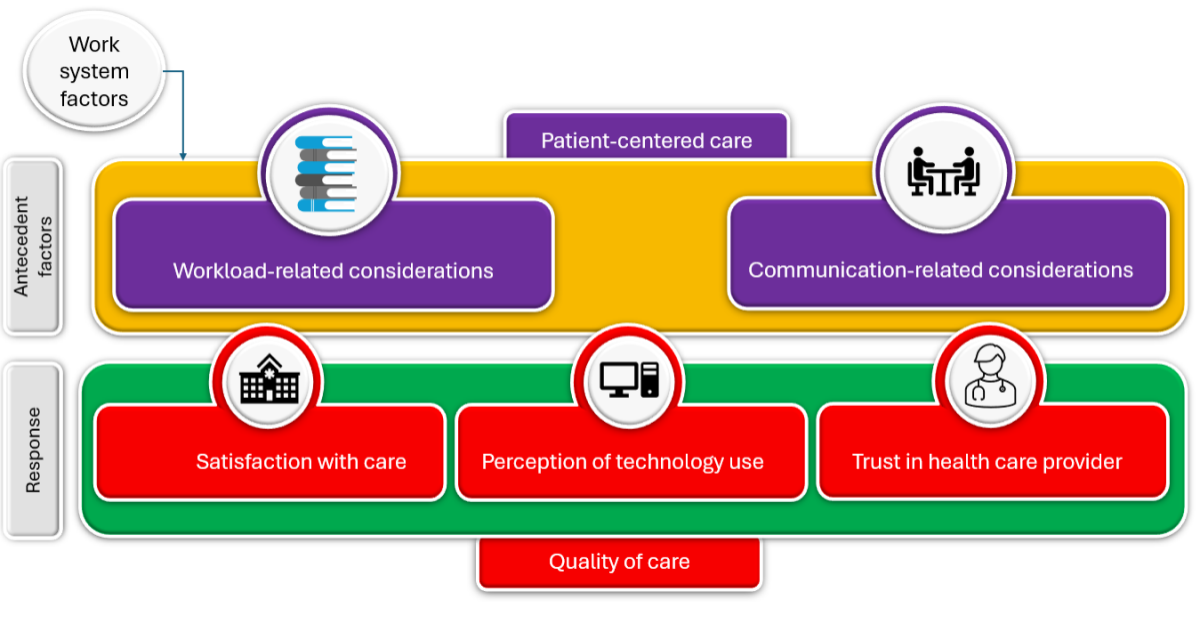
Effectiveness of Patient-Centered Cancer Care framework.

Our framework in Figure 1 emphasizes the relationship between patients’ cognitive perceptions of patient-centeredness and QOC. We account for the impact of work system factors on these perceptions. We define patient-centeredness as a combination of workload support and communication and interrelationship support. Workload-related consideration characterizes the effective engagement of patients in their care experience. Communication and interrelationship improvement describes the communication effectiveness between patients and their providers. The dependent variables are related to the action tendency of patients: *satisfaction*, *perception of technology*, and *trust*. Exposure to the work system is considered a covariate in the model. To unpack this conceptual framework for evaluating patient-centeredness effectiveness, each independent variable has operational precedent in the human cognitive factors and behavioral economics literature.

#### Patient-Centeredness (Perceptional Cognitive Input)

Effective communication with patients with cancer can help meet information needs, improve physical and mental health, promote intimacy, and reduce burden [23]. In addition, patients diagnosed with cancer spend a lot of time and effort receiving treatment. Sometimes, patients have to deal with complex tasks related to medication taking and treatment in addition to rehabilitation activities that exceed their abilities, which engenders an overburden that has been proven to cause problems with adherence to treatment plans [24]. We define patient-centeredness in this conceptual framework as workload-related consideration and communication and interrelationship-related considerations.

#### Workload-Related Considerations (Ensuring Effective Engagement and Task Load Improvement)

Patient ergonomics is the application of human factors or related disciplines to study and improve patients’ and nonprofessionals’ performance of effortful work activities in pursuit of health goals [16,25]. A central emerging concept of societal views of health care considers that patients actively perform “work” to achieve health-related goals and objectives [26]. This way, human factors position patients at the center of the work system, aiming to improve their experience with the workload assigned [25,27]. In highly sensitive situations such as cancer care, this paradigm can help us better understand the dynamics among the 3 actors of the visits (physician, patient, and technology) and how their interaction can influence critical outcomes such as QOC, trust of physicians, and acceptability and perception of technology use. We define the role of patient-centeredness as a booster to the effective engagement and performance of patients in their care through *task load improvement perception*. Thus, the effectiveness is measured through task load improvement. Cognitive task load or workload is used in human factors or organizational psychology. It operationally refers to the levels of difficulty that an individual encounters during the performance of a task and is a measure of human performance [28]. Subjective methods commonly used in research include rating perceived task difficulty, engagement, or effort made by research participants [29]. There are 3 types of workload measurement: physiological, performance based, and subjective [30]. The physiological workload measures concern the continuous size of the body’s physical responses [30].

#### Communication-Related Considerations (Communication and Interrelationship Improvement)

Compared to other health care settings, communicating information during oncology visits, especially initial ones, is critically important but can be particularly challenging due to the substantial amount of information provided, complex treatment decision options, involvement of multiple different providers (surgical, medical, and radiation oncology), and highly emotional situation with high patient workload [31]. Patients might not recall information accurately and might face difficulties understanding the information given. When information is particularly upsetting, many patients are too stunned to register further information [32]. Patients report leaving initial visits feeling that their informational needs (particularly about treatment, side effects, and prognosis) are not always met [32], which can lead to uncertainty, anxiety, and depression [31]. In one study with newly diagnosed patient with cancer–oncologist dyads, agreement on the content of the topics discussed ranged from only 37.5% for treatment side effects to 60% for prognosis [33]. Incomplete or inaccurate information about the disease process and treatment options increases the likelihood of patients receiving a suboptimal QOC [34]. Misunderstanding resulting from lack of communication has impacted health care outcomes such as decision-making, trust, and effective treatment [35]. Many countries have opened their accreditation, certification, and quality improvement programs for the past decade to examine physicians in training and communication skills [36]. Interpersonal and communication skills are 1 of the 6 general competencies for physicians identified by the Accreditation Council for Graduate Medical Education and the American Board of Medical Specialties in the United States [37,38]. “While communication skills are specific tasks and behaviors performed by individuals, interpersonal skills are relationship-oriented and process-driven, as noted by Duffy and colleagues” [39].

#### Response (QOC Perception)

Emotional distress is an average expected reaction to a cancer diagnosis. The diagnosis causes psychiatric complications (eg, anxiety, stress, and depression) induced by the patient’s perceptions of the stigma commonly attached to cancer [40]. However, it is widely recognized that patient-centered interactions have the potential to influence patients’ behavior and well-being [41-45]. Thus, we model patient-centeredness as an influencer of the behavior, which is patients’ perception of QOC (*satisfaction* with the care offered, *perception of health IT* *use*, and *trust* in health care providers).

#### QOC Perception: Perception of Health IT Use

It has been long promoted that health ITs (HITs) will improve efficiency and QOC, support health care delivery, and reduce costs for the health care industry [46]. Much of the work has assessed how health care providers and organizations can use HITs to deliver health care services [47,48]. However, a growing awareness exists that consumers also want to participate in their health care [49]. For chronic disease settings such as oncology, patients must participate in the monitoring and managing of chronic diseases [50]. Several factors contribute to the widespread use of eHealth in chronic care; acceptance and capability of using ITs are vital components of understanding the disease and treatment options [51]. Advancements in digital communication and medical technologies have led to digitalizing health care [52,53]. The increasing adoption of various HITs has created new channels for physician-patient communication beyond the walls of physicians’ offices. With the increased adoption and use rate of electronic health records in cancer care, oncologists can use the provided data in the critical decision-making process and support their workload [54]. In a study by Mazur et al [55], the enhancement of electronic health record systems’ usability was associated with better oncologist cognitive workload and performance. However, little attention has been paid to technology support for newly diagnosed patients with cancer. Therefore, extending the existing knowledge base is essential to better understand how technology impacts newly diagnosed patients with cancer. Research on the mechanism of patient-centeredness shows that it is necessary to ensure patients’ engagement with their health and their providers over the treatment time [56] as it impacts patients’ lifestyles, quality of life, and behavior in the context of cancer care.

#### QOC Perception: Trust in Health Care Providers

Extensive literature supports the importance of trust in physicians for patients with cancer as it has been linked to improving QOC and other treatment outcomes such as adherence to treatment [57]. On the basis of a review by Hillen et al [57], trust is needed to ensure a good interaction between physicians and patients. Trust has also been shown to be impacted by communication among newly diagnosed patients with cancer [58]. Thus, we consider trust as one of the QOC factors affected by the communication and workload of newly diagnosed patients with cancer.

#### QOC Perception: Satisfaction With Care

Patients demand excellent care services from their providers. It is becoming a competitive edge in health care to control the quality outcomes and patients’ satisfaction with the services, the providers, and the organizations in which they receive care [59]. Satisfaction is an outcome of utmost importance in cancer care [60]. It was shown to be related to physicians’ ability to elicit the concerns of patients’ with cancer, consider their psychosocial needs, and involve them in treatment decision-making, which are the techniques of “patient-centered” care and communication [60,61]. We consider satisfaction with care to be the third main component of the framework.

### Hypotheses and Tests

Effective communication can prevent lapses in QOC and can mitigate harm when problems occur [62]. In cancer care, it is even more important to provide patients with the suitable communication needed [63]. Improving communication with patients with cancer in the first few visits requires a better understanding of patients’ experiences of breakdowns in care and their needs in the early stage of their experiences [64]. In addition, patients frequently experience high load and feel overwhelmed due to their confusion about the treatment plans and their uncertainties about their options, which compromises their perception of QOC [34].

**Figure 2:**
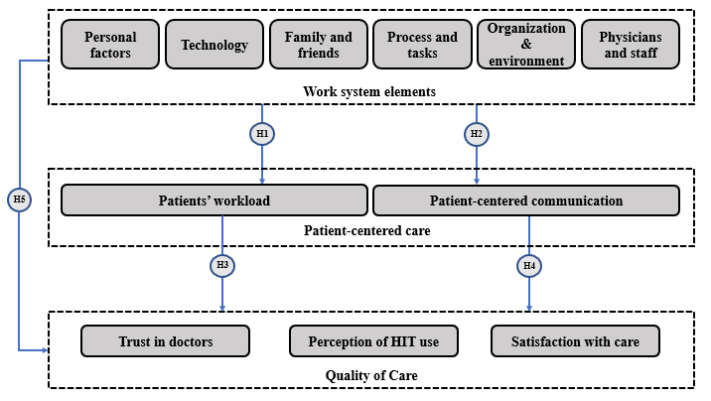
Hypothesis framework guiding the qualitative analysis. H: hypothesis; HIT: health IT.

The complexity of cancer care, typified by the financial, emotional, and physical challenges, makes patient care challenging [65,66]. In addition, the complexity of the cancer care work system is reflected in the multiple clinicians that are involved in the processes, the long therapies, and the uncertainty of the outcomes [66]. Thus, we considered the following hypotheses: (1) work system elements—work system factors impact newly diagnosed patients with cancer’s perception of their workload (hypothesis 1); (2) work system elements (communication)—work system factors impact newly diagnosed patients with cancer’s perception of their communication with physicians (hypothesis 2); (3) workload (QOC)—workload impacts newly diagnosed patients with cancer’s perception of QOC (trust in physicians, satisfaction with care, and perception of HIT use; hypothesis 3); (4) communication (QOC)—physician-patient communication impacts newly diagnosed patients with cancer’s perception of QOC (trust in physicians, satisfaction with care, and perception of HIT use; hypothesis 4); and (5) work system elements (QOC)—work system factors impact newly diagnosed patients with cancer’s perception of QOC (trust in physicians, satisfaction with care, and perception of HIT use; hypothesis 5);

We consider the following as work system factors: physicians and staff, organization and environment, family and friends, and processes and tasks. All hypotheses are summarized in Figure 2.

## Methods

### Overview

This study used qualitative data from semistructured interviews to explore the impact of work system factors on newly diagnosed patients with cancer’s perceptions of PCC and QOC, as well as the impact of PCC on QOC and technology preferences. We used semistructured interviews to facilitate candid disclosure of personal experiences. The interview questions used in this study were guided by the SEIPS 2.0 mode [26] and validated by existing literature. The full interview guide has been published elsewhere [67]. The SEIPS 2.0 model provides a framework that helps comprehend the work system (people, tools and technologies, tasks, working environment, and organization); process (clinical process, patient outcome, and organizational outcome) in the health care domain [26]. It also helps assess and understand the complex interaction among work system elements [26]. The interview guide was developed iteratively with the research team. Subsequent revisions of the interview guide were informed by emerging themes and sensitizing concepts generated through data collection and analysis. We also revised the interview guide questions based on expert feedback and the results of quantitative research conducted previously in the same center as part of the same project. Our sample included patients who (1) had been recently diagnosed with cancer, (2) were in their first few visits to the cancer center where the study took place, and (3) were adults aged ≥18 years.

We conducted a total of 20 in-depth semistructured interviews. Sampling continued until theoretical saturation was achieved, defined as the point at which further interviews did not advance the conceptual depth of the developed categories or reveal new dimensions of the relationships among categories [68,69]. The interviews lasted approximately between 30 and 60 minutes and were facilitated by a trained expert in clinical research management. The length of each interview was determined by the patient’s level of comfort in disclosing their perceptions and sharing their experiences. We completed 20 interviews, resulting in 989 minutes of recording that were used for data analysis. All participants provided informed consent for the interviews to be audiotaped and professionally transcribed.

Analysis of interview transcripts was iterative and used a deductive and inductive approach. The deductive approach used focused coding, applying predetermined codes or themes resulting from the preset hypotheses made regarding the different interactions among the perceptions of work system factors, QOC, and patient-centeredness. Throughout the study, we incorporated memo writing to reflect on individual cases, interview settings, participants’ responses, and emerging concepts and assess preconceived notions that were discussed weekly with the research team. The coding was done and visualized using a Microsoft Excel (Microsoft Corp) spreadsheet. We also prepared the COREQ (Consolidated Criteria for Reporting Qualitative Research) checklist and provided it in a supplementary file (Multimedia Appendix 1).

To mitigate the risk of bias caused by qualitative research, our study initially used a triangulation design with findings reported in different studies [3]. For more transparency and accuracy, different participants with different backgrounds reviewed and confirmed the transcripts and interpretations. In addition, a clear documentation of the analytical process was conducted.

### Ethics Approval

This study received ethics approval (institutional review board ID 00011536) from the Stevens Institute of Technology and from Hackensack Meridian Health, John Theurer Cancer Center, New Jersey, where it took place.

## Results

### Overview

The distribution of demographics is shown in Table 1. Most participants were female (12/17, 71%), White (9/17, 53%), and aged >40 years (15/17, 88%).

**Table 1:** Sociodemographic characteristics of the study sample (N=17).

			Participants, n (%)
**Gender**			
	Male	5 (29)	
	Female	12 (71)	
**Race**			
	Black	2 (12)	
	Hispanic	4 (24)	
	White	9 (53)	
	Other	2 (12)	
**Age (years)**			
	18-39	2 (12)	
	40-59	10 (59)	
	≥60	5 (29)	
**Education**			
	High school or lower	7 (41)	
	Bachelor’s degree level	5 (29)	
	Graduate school	5 (29)	

### Impact of PCC on QOC

This section presents the findings related to the testing of hypotheses 3 and 4.

#### Workload of Newly Diagnosed Patients With Cancer

Newly diagnosed patients with cancer expressed their perception of the workload and reported experiencing a high mental workload due to frustration and emotions when they were first diagnosed:

At first you’re all nervous and upset with your condition. So you’re like, I don’t know how to do this. You’re freaking out.

Patient 13

The mental workload increases as patients are required to remember many details (eg, appointments, information, and medication) and are required to understand the results and options they are given with the little information shared with them:

There’s a lot to remember. There’s a lot you got to remember which medicine to take and when.

Patient 12

I think sometimes the interpretation of the test results is challenging and a bit anxiety producing.

Patient 06

In addition, patients experience high temporal and physical load as their tasks require a lot of effort and are time and energy consuming:

It’s unfortunate that I have to get drawn so many time...You just get tired of sitting, you get tired of being in there.

Patient 02

Very demanding. I go get the blood work done first, and then when my blood work is done, I go up and wait to see the doctor. Then I go to the infusion room when I’m done with him.

Patient 13

#### Impact of Workload on Trust in Physicians

High workload negatively impacts newly diagnosed patients with cancer’s trust in their physicians. For instance, patients who had less workload, felt cared about, were given enough time, or were less rushed were more likely to trust their physicians:

My doctor really care about me. I gage that because I’m not being rushed to leave right away. I see the attention that they pay attention to all my questions that I have, and also, I feel comfortable.

Patient 03

I think they do help with my emotions because they are trying to address every question that I have. I am not getting very emotional; I’m not getting extremely upset. So what I’m trying to say is that they might not even see any emotional reactions from me because I do trust that the doctor said he will fix me and I know that he will. So, I’m not emotional. I’m going very strong because of their support and my knowledge that they are there, and they will fix the situation.

Patient 05

#### Impact of Workload on Satisfaction With Care

The findings also showed that the workload perception among newly diagnosed patients with cancer impacts their satisfaction with the care services received:

As far as the demands, the time and effort it took to ensure the entirety of the visit was completed well in its entirety. Look, it was very effortless on my part.

Patient 06

They just gave you time to digest and let you just sit there and think about what the doctor was saying, and that really does make a difference. So basing out the information across the appointment instead of just kind of ramming it all down your throat at the beginning and asking any questions, it always moves quickly. But I do think that the taking of time in between providing information and providing time to digest was very helpful.

Patient 12

However, patients who felt that the care services were demanding of time or effort were less satisfied with their visits:

The treatment itself, when I’m there for chemo, does take a long time. That’s like 4 hours at least. And then I’m on a pump for the next two days, so that does take a decent amount of my time. And then when I’m on the pump, I’m basically laying down because I really can’t do much because I’m still getting treated. So, the treatments are demanding.

Patient 13

#### Impact of Workload on Perception of Technology Use

Finally, the high workload impacts patients’ approval of physicians using technology during the visits. Patients who felt that using technology made their tasks less effort, memory, and time consuming were more likely to accept it. They felt that having all the information available in one place saved them from looking for information everywhere and trying to understand it and share it again with the physicians:

Technology can help keep me cooperating with the doctors and the treatment without much effort...Usually the nurse has the computer and she’s checking in that all the details are correct and verifying information with me before the doctor even walks into the room. And she can answer questions about when I start the next cycle, et cetera. Because she has all the information with her. That saves me time. I don’t need to remember everything.

Patient 02

It will impact only positively because I see an order, everything in one place in my app. I see my history, I see all my tests, I see all my appointments, all notes from the doctor, I see all the scans and everything is in one place. For me it’s positive because again, I’m technology savvy and for me, that’s great to be utilized, to have everything in one place.

Patient 05

To conclude, these qualitative findings showed that having less workload (eg, physical, temporal, and effort based) helped newly diagnosed patients with cancer trust their physicians more, be more satisfied with the care visits, and accept technology use during the visits (hypothesis 3 was supported).

#### Communication Needs and Impact of Communication on Trust in Physicians

The quality of the communication in cancer visits is critical. Physicians share a lot of information with patients in the first visit, and it is essential for them that physicians explain things clearly in an easy, comforting, and understandable way and that their questions are answered without being rushed. Good communication of the information needed made patients trust their physicians:

She made it very easy for me to understand very difficult, very complex procedures and explain to me clearly why, in my particular situation, she would recommend the clinical trial treatment that I’m on. And the appointment didn’t last more than maybe 20 minutes, but in that time, I felt like I understood what I was getting myself into. She provided things for me to read when I got home and explained clearly that other options were not as good as this one...If I was concerned about something, she would try to reinforce that. Everything that she explained made sense because she was trying to keep me at ease and not worried about more than I should have.

Patient 02

Our communication me and the doctor is genuine, accessible, nurturing, informative.

Patient 17

Different patients also have different levels of understanding. Patients trust their physicians if they respect their pace and health literacy levels:

Yeah, basically pausing and making sure I understand things as she’s saying them, making sure I’m caught up in the discussion, because you could go very fast over a lot of things and there’s a lot of information to digest at an appointment like that. So she would take her time to pause and say, do you understand what I’m talking about? So that sort of thing helped.

Patient 17

Another important communication factor that impacted patients’ trust in their physicians was feeling like they were treated equally without any bias independent of the type of insurance they had or their age or race. Patients liked to be treated as human beings, not as numbers:

It felt good. I didn’t feel like I was going to be just thrown in there and just done whatever, and then no explanation of anything, so it felt really good. And the mere fact that I have Medicaid and I didn’t feel like a patient, that felt phenomenal for me. It was very powerful. Well, I mean, it seems that we get lost in the shuffle. We’re just like, nobody basically. We seem to be like, I don’t know, nobody, we’re not treated like we just don’t matter. I didn’t feel that way at all. I felt like I have a chance just like everybody else who has like [insurance name] or whatever. So I just felt like they cared about me and the process was they were going to do whatever they had to do, no matter what insurance I had. So that meant a lot to me.

Patient 07

Furthermore, newly diagnosed patients with cancer like to be given full attention by their physicians, communicating both verbally and nonverbally, exchanging eye contact, and paying enough attention to every question they ask. This impacts their trust in physicians:

Well, they always examine me, obviously. They talk to me. They come up to me, and they look at me not everywhere in the room, just looking at me eye to eye, and explain to me exactly what happened this week...They look at me straight in the eyes, and for the time that we’re there, her attention is focused on me. And when I ask the question, they usually don’t mind repeating themselves, because sometimes when you’re in treatment, you don’t hear well. And I’m taking notes when I’m there. And I sometimes repeat questions that she may have already answered. And she is very happy to follow up and expand a little more so that I can understand in more detail what she’s trying to tell me about follow up questions, answering follow up questions.

Patient 02

Finally, patients want to be treated in a personalized way as a special person and to build a strong relationship based on empathy with their physicians by talking about their personal life and not only about treatment and visits:

My doctor is extremely approachable despite his busy schedule. He shared his cell phone number with me, but of course I’m not going to communicate to him. But those are things that you understand that you’re not just a patient, you’re a special individual for him and for his staff, and everything is personalized. I think that’s my belief, honestly.

Patient 05

I like that the doctor talks to me in general not only about my treatment. Sometimes we talk about family and things like that, really getting to know the doctor. He was very communicative; he keeps being positive and that helps.

Patient 01

I think in particular, she has quite a good amount of empathy, which I think a lot of doctors don’t. So she treats you like a human being and trying to think what else on the medical side. It’s basically kind of explaining your condition well and giving you an idea of what’s to come.

Patient 10

In fact, patients who do not have a strong bond with their physicians and only talk about treatments without discussing their options may have low trust in the physicians’ opinion and feel that they are treated in an unfair way:

Well, yeah, I feel that, but I think this is more and more interested in selling products that are profitable for the hospital than necessarily what care I need. She just seems obsessed with selling an expensive procedure that I’m not ready for. I’d like to see more programs tailored to my situation and some options. There’s a lot of options and treatments available today. And to say it’s this or nothing, I don’t think it’s appropriate.

Patient 11

#### Impact of Communication on Satisfaction With Care

The communication between newly diagnosed patients with cancer and their physicians also impacts their satisfaction with the care received during the visits. In fact, patients prefer to be told the truth about what is happening to them and for it to be stated that the physicians are doing their best:

Well, anything the doctor noticed, any concerns she has, she always meant that she just wants to be sure and just saying that she wanted to stay on top of things and that was pretty good to me. I would say I had really good care both in first visit and follow-up ones.

Patient 01

They also want physicians to explain the goal of the visit clearly and to be walked through every step of the procedures. On the basis of patients’ needs, physicians need to ask them whether they are satisfied or not and allow families to be part of the visit for more support:

When I have to come visit, they know exactly what happened before, and they can specifically tell me where we are today and what we need to do today. They also keep some time for me to ask questions, and if I don’t understand something that she explained, she’ll explain it again. My daughter participates in those visits as well, and she always has questions too. As long as we have questions. If we are all clear and everything’s just a routine visit, then we need less time, I guess. But if we need more time to answer questions, they’re willing to answer until we’re satisfied...I like it when they explain things to me, even if they’re technical, because I can look it up and kind of make sure that I’m getting the right information from my doctors and it’s consistent with what the best health organizations treating cancer are doing.

Patient 02

Although some prefer to be told everything, others do not understand much when it is too technical. Physicians need to explain things to them in an understandable way:

The communication was excellent. The answers that I was seeking were given and the questions that I had were answered. The care was given, felt comfortable. The doctor communicated with me in a way I could understand.

Patient 06

However, paying attention to special cases is important to gain patients’ satisfaction. Some patients have comorbidities and need to be taken care of in a more careful way. For example, one of the patients whom we interviewed was blind. It is more challenging to communicate with such patients:

I am blind, so they always print documents out for me though but they tell me everything verbally. To make sure I understand and then they tell me it’s on the printout.

Patient 16

Some other patients are skeptical about health care systems. It is important to know how to handle them and how to communicate information to them without losing their trust not only in physicians but also in the system itself:

Cancer care is a profit center for these medical centers. The doctor is trying to push a very expensive procedure that’s very invasive that I don’t have the support network to do. And it seems to be like she wants an all or nothing for that procedure. So, I think I really need a second opinion on this stuff. Well, like I said, I think that the cancer centers seem to be out to maximize profits because I see them advertised all over. I don’t know, that’s just what I seem to find out.

Patient 11

Finally, despite the focus on visits being very important, follow-up needs to cover home care for the first visits as patients need more support at the beginning of their experience and building a bridge of communication with them beyond the care visits would help them feel more cared about:

So, the communication during the treatment and while I am in the hospital is really good and I feel that I can ask any question and I always get the answers. As for communication when I am at home, I think I am still learning the system. After my first treatment, I had some adverse effects. I did document and I did write up my observations. Science in me did that. But I didn’t know how to communicate that to the doctor until my next follow up with the doctor. And then we did discuss those adverse effects and they did adjust my dosing regimen.

Patient 05

#### Impact of Communication on Perception of Technology Use

Newly diagnosed patients with cancer need their physicians to communicate with them without any distractions:

No, no them using a computer is of no distraction at all. The doctors still attend to me.... They always check my blood work and put it on the computer and if there is anything they communicate after the visit. They always call.

Patient 01

If patients are made the center of the visits and the computer is used for documentation purposes, patients feel satisfied with its use by physicians:

I don’t think technology is disturbing my communication because they are still there. It’s not that we are communicating through computers only. They are still there in the room they are personally discussing with you. But then they document everything in the computer. And I think at this time and era, you do expect that everything will be documented on the computer? That’s my expectation.

Patient 05

She occasionally doesn’t always use the computer, but occasionally does to look at test results. But I never really found it distracting, and I don’t feel like she was paying attention to the computer more than me. It was just there as a tool as part of the appointment. Never did I feel like it was computer first, patient second sort of thing.

Patient 12

However, if the use of technology made patients feel that they could not communicate well with their physician, they were not happy with it being used during the visits:

Not every time, but yeah it was distracting, sometimes. Actually, now that I think about it, I think that was where I could see a few instances where that was where their focus was. I think I would ask a question and there would be like a two-minute pause because he was in the middle of typing stuff on the computer and then he would answer after. So, the doctors was distracted with whatever he was doing on the computer.

Patient 08

I would prefer them not to be on their computer and rather making eye contact and communicating directly with the patient rather than typing. So I didn’t feel as connected with the doctor.

Patient 17

To conclude, these findings showed that, if the communication between physicians and patients is built in such a way that patients are the center of the care and using technology does not distract physicians from building a bond with their patients, technology use during the visits is accepted and not judged as distracting. Thus, hypothesis 4 is supported.

#### Impact of Work System Factors on Communication

The work system factors in health care impact patients’ communication with physicians. In fact, patients were more satisfied with the communication with health care staff when they felt that the organization was empowering nurses to intervene and raise issues related to their health. Thus, the *organization and environment* impact the communication perception among newly diagnosed patients with cancer:

There was a nurse, also a night nurse, who noticed that there was fluid in my lung and she put a note in for the doctor to see if they could remove it because she thought it was too big. The day after I got in from the emergency room and the nurse was the one who raised the flag to the doctors and the next day they removed the fluid. So I think they’re empowered, but at the same time looking she didn’t have to go back and look at the X rays in my lung because she was surprised that I was breathing so badly. So, she was just curious and checked in and brought it to everyone’s attention. I also like that they called me directly to check on me. They make sure that we are cared about and that we know everything about our situation.

Patient 02

In addition, frequent follow-ups can help patients share their concerns and issues with their physicians and help them communicate well in a continued way, which shows the impact of the *processes and tasks* on communication for newly diagnosed patients with cancer:

So, I think follow-ups are very important, especially after the first treatment. When my first treatment was very miserable, I felt very miserable afterwards, I had very significant adverse effects to the drugs. And then in a week I had follow up with the doctor and we had really good communications for the second treatment.

Patient 05

The way in which physicians, nurses, and staff interact with patients impacts their perception of communication. For instance, patients were more satisfied with physicians and other staff if they felt cared about and if their questions were answered. In addition, allowing family members to attend visits may help patients feel more reassured:

The people in the lab are amazing. They understand that we get pinched a lot, and they try to work with you, and they help each other, too, because they have to get the results stat, how they like to say. And they look thoroughly at the request from the doctor. And if I have questions about what they’re doing, they’ll answer them intelligently...When I first saw my doctor, she knew the record just as well, but she asked me to tell her my experience so she would know firsthand from me how I was feeling now and what had happened in the past. So, I felt very well taken care of and the communication between my doctor and me was excellent really.

Patient 02

My daughter participates in those visits as well, and she always has questions too.

Patient 02

Thus, we conclude that the work system elements impact the perception of newly diagnosed patients with cancer regarding communication, which supports hypothesis 2.

#### Impact of Work System Factors on the Workload

The work system elements impact patients’ workload. In fact, the process of detailed documentation in the records and providers accessing that information easily also reduces patients’ mental workload and frustration. Patients also like being guided through every step at the clinic as it helps them feel better:

I do feel supported, even though we meet for short periods of time...I felt that in every visit, in the few minutes that she was maybe 15 minutes that she’s in the room, she knows everything about what happened the previous weeks. I don’t know how she does it, but if I forget to tell her about something that I was feeling the week before, she would ask me about it. So, I understand. However, they do it to go into the room and remember exactly this patient in particular, it makes me feel very reassured that they’ve done their homework when they walk into the room to talk to me.... I think they have a pretty good system. Once you register, they have someone already greeting you, walking you over to do any lab tests that you need. They kind of wait around and guide you to the elevator so that you can go up to the waiting room waiting area.

Patient 02

You come and you get greeted by a person that sits on the first floor. Then you go to lab. In the lab everyone is very attentive. Sometimes you have to wait a couple of minutes, but usually it’s not very long wait time and they are very attentive to ensure that they are doing very good.

Patient 05

However, the long waiting time in the process makes patients anxious and nervous, which adds more workload to the physically demanding processes and procedures that they are experiencing:

I just wait in between seeing the nurse and the doctor a little bit too long, I thought. So, the wait was a bit lengthy, a bit long. That was nervous for me. And not only that, but we had to leave at a certain time because we had to go pick up my nephew after school. So that was my appointment was at I think it was at 110, and I didn’t see the doctor to, like, almost 230 or something like that.

Patient 07

I sat there, and I waited, and I waited, and I waited for my first biopsy results and to get them at 02:00 p.m. I was calling and calling and calling, trying to see if anything came in...It’s a lot and it takes a toll on you.

Patient 13

To reduce this load, physicians and staff members need to explain matters clearly to their patients to comfort them, reassure them, and make them feel cared about through personalized services:

I would say the doctor knew how much information I needed to avoid being overwhelmed. Just telling me the options of what we need to do and I think that pretty much helped. Not feeling overwhelmed, it’s like, let’s do this and get past it. So that was pretty much my feelings.

Patient 06

I was very nervous about what the nurse had told me that was going to happen once. I didn’t want to need to have a tube in my lungs. But luckily, before we got to the procedure, they had already taken care of that and she put it in capital letters so that the radiologist didn’t miss it, that they didn’t need to put it too, because the treatment would resolve that over time. So, I think reassurance is what they tried to do and being attentive to the details, which in medicine, I think is very important because each case is a different case. And I felt very comforted that I’m not just a number, I’m a patient that they’re trying to get out of the hospital.

Patient 02

What I do when I am overwhelmed is I call the nurses all the time, and they’re so helpful. I was calling them multiple times a week, and whether it was a new side effect, or I just had follow-up questions. So, I definitely have been utilizing them, and they’ve been so incredibly helpful.

Patient 08

In addition to the health care actors, the organization impacts patients’ workload. Patients need a relaxing, calm, clean, and organized environment. Using comforting colors and decorative signs that motivate patients can also give them more hope and reduce their load:

So, when we look at the physical layout of it and all those processes, it’s very nice, very organized place, very relaxing when you have to wait, so it’s no problem.... Everything was very comfortable. No noise at all. Very calm and especially very accommodating.

Patient 01

The environment is really good. Everything flows nicely. Everything is nice and clean. Everything the colors and the walls and everything is very calming. As far as decorations and stuff like that, there’s like a passageway that sometimes I’ll pass through that I see like puzzles of past people that I have done from cancer. They do like these jigsaw puzzles, and they’ve hung them all across this hallway that I pass, which I find very endearing when I pass, and I see that. So pretty much in the sitting areas and everything where everyone sits and waits nice, valid and everything like that.

Patient 03

Construction work, long walking distances between the rooms, parking, and many other issues may cause patients to experience more physical and temporal load:

Let’s be honest here. Like, it’s completely overbooked, and I know they’re under construction or what have you, which is stressful to have that many people crowding in the hallway.... Like if I was upstairs and I couldn’t make it down in time, they would call and say, hey, she’s running late. And it was accommodated. But it’s definitely overwhelming to navigate.

Patient 17

You can build a bigger parking lot if you have room. Yesterday, yeah, I was riding because the appointment was at 01:00 at that time was all packed. They had to drive around all the way up to the roof and start coming down...there are definitely not enough spots there.

Patient 09

Furthermore, family and friends can help support patients in their tasks, which reduces their workload by facilitating processes:

My daughter is there for me. I moved in with her so that she could drive me to my appointments, and I can have support when I’m not feeling so good.... It makes things way easier.

Patient 02

Wife, family, friends, taking me to the treatment, stopping by and visiting me, phone calls. Just a lot of support.

Patient 08

My husband is always next to me. I am 90% self-cared, but sometimes I need his help to move around. For example, during the chemo to go to the bathroom.

Patient 05

On the basis of these findings, we showed that work system elements impact patients’ workload. Thus, hypothesis 1 is supported.

#### Impact of Work System Factors on Satisfaction With Care

The work system elements impact patients’ perceptions of QOC. They impact patients’ trust in their physicians, their perception of HIT use, and the satisfaction with the care received during the visits. In fact, patients appreciated nurses checking on them frequently and being nice to them, which made them feel more satisfied with the care received:

I think specifically the nurses in the infusion center, they were so kind and so nice, and they definitely were always asking how I was feeling during the infusions. They come check on me every ten minutes, pretty much. So, they were very accommodating and made me feel very comfortable.

Patient 08

Before seeing the doctor, you see the nurse nurses always welcoming even the staff that you go for copay or just approach to announce that you arrived. They are very attentive. You can see that they are feeling your pain. And that’s very comforting, let’s say...Usually my chemo is very long. So, the people who delivers lunch are so attentive to every single person. They are taking time for every single patient to repeat whole menu and convince you that this is very delicious to take. It’s really warm and nice atmosphere. So I think they are taking extra steps to make you feel as comfortable as possible given the heaviness of the disease.

Patient 05

Receiving less attention from physicians would make patients unsatisfied with the visits:

My doctor is very busy. He’s the head of the cancer center and he has tons of patients. But I would have liked to see him maybe sometimes not at the end of the treatment, but also in the middle of the treatment.

Patient 05

In addition, having a good organizational environment increases patients’ satisfaction with care. Patients want to be in a well-designed environment where they can access better care services:

So, I think all is very accessible, very well designed, that you stop by first in the lab, then you can immediately pick up your pharmacy needs and then go to the second floor to visit the doctor and then move to the chemo center. So, I think everything was designed well. I love the sunny side and shady side of the infusion room. They have all these blankets, very nice people always asking what you want, more water, more anything to make you feel better. I think, as you say, from the organizational and structural perspective, is designed very well.

Patient 05

#### Impact of Work System Factors on Trust in Physicians and Staff

When physicians provide them with personalized services that are not based on generic information and that speak to their needs and situations, patients tend to trust them more:

It’s just from reading the reports that the doctor gives me after the visit summary, I can tell that it’s not generic. It’s definitely speaking to my condition. I can see that what they’re writing, and my evaluations are definitely about me, and I can see the reports, and it’s definitely very much personalized.

Patient 12

The mix of personal and professional interaction makes them trust their physicians and the nurses delivering the services. Patients need people who listen to them, a friendly environment, and practices in which the main goal is to deliver the safest care to them:

What I did notice you have very good clinical practices that when the nurse has to introduce chemotherapy, they have second pair of eyes verification, which speaks of high quality and regulatory compliance of your organization, and that is incredible to see.

Patient 05

Every time I have a discrepancy, they always double-check. Either the nurse in the infusion room double-checks with the research nurse, the research nurse double-checks with the doctor, and everybody double-checks to make sure that we’re doing it the right way, and that gives me comfort as well.

Patient 19

I think it’s a good team. They listened to their own people, and they acted on it. That makes me trust the whole thing. So, I’m like I said, very professional and very personable. I really like that. It sums it up so beautifully. Like the two pieces in health care. Professional yet personable. I really do like that.... The hospital has great practice, and we have a number we can call. Twenty-four, seven. And they told us exactly how to behave and where to go when we got to the hospital. So that kept me more of these. But I was very scared. It was a Sunday night, so the doctor didn’t physically come, but the emergency doctor had spoken to her. He knew my case.

Patient 02

They had a social worker contact me, which was nice to see if I had any opportunities or anything. I thought that was a good guess here. Any helping hand is an essential hand.

Patient 11

In addition, more trust is built among patients if the team’s communication is healthy and professional:

The communication between the staff, I see that it is good and very professional. And the place is amazing. They walk with me and make sure I have all what I need to start the next step. The infusion, I prefer the one where I get sun and they already know that.

Patient 02

#### Impact of Work System Factors on Technology Perception

If physicians let themselves be distracted during the visits or do not pay enough attention to patients, patients may consider technology as a source of distraction and disruption to the visit:

The doctors was distracted with whatever he was doing on the computer.

Patient 08

Finally, the organized process of the visit, the good collaboration between nurses and physicians, and note-taking to make patients the center of the visits made patients trust their physicians more, be more satisfied with the visits, and accept the potential that technology may have in the success of the care processes:

Doctor showed up even with nurse practitioner. And normally the doctor talks to you, she examines you, explains things. And then normally the nurse practitioner fills up whatever they need to do in a database. And normally it’s not distracting at all. It’s all adequately it happens in the background, and you concentrate on the conversation with a doctor, and someone else is filling out all the paperwork. Something needs to be like sending the prescriptions to my pharmacy and setting up another test. Everything was done at the same time, but I don’t feel it was destructive at all. It was good.

Patient 09

Thus, to sum up, the health system elements impact patients’ perception of technology use, trust in physicians, and satisfaction with care, which supports hypothesis 5.

## Discussion

### Principal Findings

In this study, we qualitatively explored the impact of work system elements on QOC and PCC and how PCC also impacts QOC among newly diagnosed patients with cancer in the first follow-up visits after the diagnosis. We found that newly diagnosed patients with cancer experience a high workload (mental, physical, temporal, effort based, performance based, and emotional) resulting from the frustrating diagnosis and the load of information that they receive in the first visits. This load impacted patients’ trust in their physicians, satisfaction with care, and perception of technology use during the visits.

A diagnosis of cancer is a threat to one’s sense of security, whereas feelings and emotions accompanying the disease uproot everyday existence [70]. Patients find themselves unpredictably facing a high emotional load and under the obligation to cope with the stress and anxiety caused by their diagnosis [70,71], which explains the high emotional and mental workload faced by our participants.

In addition to that, patients with cancer have to deal not only with the physical ailments resulting from the illness and its treatment but also with the thoughts of permanent health impairment, disability, fatigue, and pain that may result from their diagnosis [72], which correlates with our finding of high physical and effort-based workload perception among the participants. This may explain the dissatisfaction of patients with the quality of the care received. Emotional stress and mental problems can cause difficulties in everyday life, such as not being able to work, financial problems, and a lack of social support. This has been shown to impact quality of life perception among patients with cancer in other studies [73]. The literature also shows that patients with cancer can experience a variety of needs as each person reacts individually to the hardships of illness depending on their personality traits and understanding of their new situation [70]. With the substantial incoming flow of information, patients may find themselves unable to trust physicians and may consider technology as a distraction to their visits at that stage.

We also found that newly diagnosed patients with cancer can be very needy when it comes to communication with their physicians and that their communication with physicians impacts their perception of QOC. Communication is the cornerstone of the relationship with the patient in all medical settings, specifically chronic care, with the main aims of creating a good interpersonal relationship, exchanging information, and making treatment-related decisions [74]. Certain attitudes, behaviors, and skills (eg, ability to impart confidence, empathy, “human touch,” relating on a personal level, being forthright, being respectful, and being thorough) are part of effective communication, which was validated by our findings in this study [74]. A poor physician-patient communication in cancer care negatively affects psychological well-being and patients’ decisions and perceptions regarding treatments. This validates our findings of the impact of communication with physicians among newly diagnosed patients with cancer on their perception of technology use during visits and trust in physicians [75].

In addition, we found that the work system elements impact patients’ workload, communication, and QOC perceptions. This correlates with the findings of other studies in which the environment design was shown to impact patients with cancer’s perception of QOC. A recent review of evidence-based design also found that a conscious design adapted to patient needs had an impact on a decrease in infection spreading, length of stay, pharmacological needs, and perceived stress among patients [76]. Furthermore, symbolic objects found in the environment have been shown to impact patients’ sense of self and well-being [76].

In addition, a recently published Cochrane review on environmental impact on health stressed the profound need for well-designed studies following intervention in health care environments [77]. This correlates with our finding that patients who liked the decoration of the hospital, the motivational signs, the colors, the cleanness, the organized processes, the lighting, and the care of the nurses were more satisfied with the QOC and felt less overwhelmed. These findings lead to the expectation that major considerations ought to be taken when designing health care environments to meet quality requirements while considering patients’ needs and supporting patients’ sense of control, autonomy, and independence.

### Theoretical and Practical Implications of the Findings

In the previous section, we validated the preset hypotheses that correlate with the findings of the quantitative studies from the greater project. This framework can help inform patient-centered interventions that aim to provide newly diagnosed patients with cancer with the support needed and ensure their satisfaction with the QOC offered. More empathy and human bond links between physicians and patients should be considered as patients want to be treated in a more patient-centered way and to feel that they are not receiving the same care as everyone else in the same way.

Patients also want to have the chance to ask as many questions as possible and be given as many follow-up visits in the beginning as possible to receive comfort and reassurance that everything will be fine. Empowering workers (nurses and staff) to intervene in case of emergency would help patients trust the health care organization. In addition, allowing patients to be accompanied by their family members would help them be emotionally comfortable. Another point to consider is to share a second screen with them in case a computer is used when the physician is communicating with them to comfort them regarding what the physicians are doing when they are not talking to them.

Pausing in the middle of the discussion to do other tasks would result in losing the patients’ attention. Physicians should consider continuous communication where they pay as much attention as possible to the patients in a friendly way and where they listen to their concerns without rushing them even if there is a time limit as the time given can influence their decision-making process importantly.

This study’s findings can also inform the organization’s design. It should be considered that patients cannot move a lot between the laboratories and the visit rooms and it would be easier to assign them to rooms that are close to each other to minimize their physical effort during the visits. Better scheduling and allocation strategies should be considered to minimize the waiting time inside the hospital for each patient. Comforting colors, relaxing decoration, and motivational signs would help reassure patients while in the hospital. In addition, having any construction work when patients are coming in and out should be avoided as that can add more load to what they are already experiencing.

### Limitations

Despite the useful insights garnered from this research, certain limitations must be addressed. First, the study’s narrow geographic reach, which included only 1 cancer center, may limit the findings’ generalizability to other cancer populations or health care settings. Patients’ experiences and technology preferences in this facility may not represent those in other cancer centers or varied communities with different demographics or cultural backgrounds. Second, selection bias is possible as patients who chose to participate in the interviews may have different characteristics or opinions from those who declined or were unavailable. This may introduce bias in the findings and reduce the study’s external validity. Furthermore, interviewing patients within a few visits following their initial diagnosis may not completely capture the dynamic character of their technological choices, which may change over time as patients adjust to their diagnosis and treatment. The reliance on patients’ recollection of their technology preferences at this early time point may also be subject to recall bias. Furthermore, contextual factors particular to the cancer center where the research was conducted, such as local health care policies and the availability and accessibility of technology, may not be applicable or may vary in different contexts. Finally, social desirability bias and interviewer prejudice throughout the data collection process may have an impact on the data’s authenticity and veracity. Despite these limitations, the findings of this study provide valuable insights into the technology preferences of newly diagnosed patients with cancer, and additional research with larger and more diverse samples, longer follow-up periods, and considerations of contextual factors is required to strengthen the findings’ generalizability and validity.

### Conclusions

In this study, we suggested a framework called Effectiveness of Patient-Centered Cancer Care and tested its validity in cancer visits to support PCC among newly diagnosed patients with cancer using qualitative data. We found that workload and patient-centered communication impact QOC and that the work system elements impact the patient-centeredness (workload and communication) and QOC (trust in physicians, satisfaction with care, and perception of technology use). To improve patients’ experiences in the first visits after diagnosis, more interest needs to be given to the design of the organization, the processes that the patients have to go through, and the collaboration among the different actors and providers. This study’s findings can also inform the organization’s design. It should be considered that patients cannot move a lot between the laboratories and the visit rooms and it would be easier to assign them to rooms that are close to each other to minimize their physical effort during the visits. Better scheduling and allocation strategies should be considered to minimize the waiting time inside the hospital for each patient. Comforting colors, relaxing decoration, and motivational signs would help reassure patients while in the hospital. In addition, having any construction work when patients are coming in and out should be avoided as that can add more load to what they are already experiencing.

## Appendix

Multimedia Appendix 1 COREQ (Consolidated Criteria for Reporting Qualitative Research) checklist.

## Abbreviations

COREQ: Consolidated Criteria for Reporting Qualitative Research
HIT: health IT
PCC: patient-centered care
QOC: quality of care
SEIPS: Systems Engineering Initiative for Patient Safety

## Data Availability

The data sets generated during and analyzed during this study are available from the corresponding author on reasonable request.
